# Growth mindset in young people awaiting treatment in a paediatric mental health service: A mixed methods pilot of a digital single-session intervention

**DOI:** 10.1177/13591045221105193

**Published:** 2022-06-01

**Authors:** Brian CF Ching, Sophie D Bennett, Nicola Morant, Isobel Heyman, Jessica L Schleider, Kate Fifield, Sophie Allen, Roz Shafran

**Affiliations:** 1 11700 UCL Great Ormond Street Institute of Child Health, London, UK; 2 4956 Great Ormond Street Hospital for Children NHS Foundation Trust, London, UK; 3 4919 UCL Division of Psychiatry, Bloomsbury, London, UK; 4 Department of Psychology, 12301 Stony Brook University, Stony Brook, NY, USA

**Keywords:** Anxiety, children, mental health, depression, digital, mixed methods, young people, growth mindset

## Abstract

**Background:**

Wait times are significant in child mental health services but may offer opportunity to promote growth mindsets in young people with physical and mental health needs. A digital growth mindset single-session intervention is effective in young people, but its use in paediatric settings has not been examined. This mixed methods pilot aimed to assess the intervention’s feasibility, acceptability, and impact in this population.

**Method:**

Patients aged 8–18 on waiting lists in a paediatric hospital’s specialist mental health service were offered the intervention remotely. Treatment completion and retention rates, symptoms of depression and anxiety, perceived control, and personality mindset were assessed at baseline, post-treatment, and follow-ups. Semi-structured interviews to explore the intervention’s acceptability were conducted post-treatment.

**Results:**

Twenty-five patients completed the intervention and 17 patients and three carers/parents were interviewed. Outcomes showed small to large improvements across time-points. Most patients reported finding the intervention enjoyable, accessible, and instilled a hope for change. They valued elements of the intervention but made suggestions for improvement.

**Conclusions:**

The digital growth mindset single-session intervention is feasible, acceptable, and potentially beneficial for young people with physical and mental health needs on waiting lists. Further research is warranted to examine its effectiveness and mechanism of change.

## Introduction

People’s beliefs include mental representations of the self (e.g., one’s personality, qualities, and traits) and are related to outcomes, such as adaptive responses to stressors (Erdley et al., 1997; Yeager et al., 2013), coping, and quality of life (Griggs & Walker, 2016) in young people. The belief that one’s attributes (such as feeling depressed or ability to cope with adversity) are malleable and can be developed is termed a ‘growth mindset’ and underlies adaptive functioning (Dweck, 2008). It is driven by perceived control, which comprises of primary control (control of objective events/conditions through behaviour) and secondary control (control of the psychological impact of such events/conditions) (Weisz et al., 2001, 2010). Young people with a growth mindset have a lower risk for depression and anxiety than those with beliefs that personality is unchangeable, a fixed mindset (Schleider et al., 2015; Schleider & Weisz, 2016a).

Young people with functional symptoms (a preferred term used to describe persistent physical symptoms without clear organic cause that impairs functioning; Marks & Hunter, 2015), are at particularly high risk of developing mental health problems, such as anxiety and depression; 71.7% of children with mental health problems also had physical symptoms (NHS Digital, 2018). Although this specific group of young people benefit from psychological treatment, including brief interventions (Bennett et al., 2015; Catanzano et al., 2020; Moore et al., 2019), reduced access to evidence-based treatments due to long waits may have negative effects on mental health outcomes (British Medical Association, 2017). There is evidence from referral data to UK child and adolescent mental health services that children with combinations of physical health needs and emotional symptoms receive low rates of intervention and follow-up (Children’s Commissioner, 2016).

Single-session interventions (SSIs) may bridge this treatment gap and offer useful input during periods of waiting for longer term treatments. An online growth mindset SSI that aims to develop adaptive growth mindset beliefs has demonstrated effectiveness in improving perceived control, stress responses, and symptoms of depression and anxiety in youth, may enhance care, and potentially improve outcomes (Miu & Yeager, 2015; Schleider & Weisz, 2016b, 2018; Schleider et al. 2021a, 2021b). However, little is known about its effectiveness in young people with both complex physical and mental health needs. Such an intervention may be particularly beneficial as reduced perceived control is associated with psychological distress in children requiring medical care (Carpenter, 1992; Hoff et al., 2002).

This pilot aimed to:

(a) Assess the feasibility and acceptability of a digital growth mindset SSI in young people on waiting lists for mental health assessment and/or treatment in a paediatric hospital, through recruitment, treatment completion, and retention rates, and qualitative interviews;
(b) Preliminarily evaluate its impact on symptoms of depression and anxiety, perceived control, and personality mindset.

## Methods

A mixed methods case series design with the principal method being quantitative that is complemented by qualitative methods (see Morgan, 1998) was used to pilot the intervention. Quantitative data were collected at baseline, post-treatment, 1-month follow-up, and 3-month follow-up to assess feasibility and preliminary impact of the intervention. Qualitative data were collected using semi-structured interviews at post-treatment to enhance understanding of the quantitative data and explore acceptability of the intervention.

### Ethics

Approval was granted by the Great Ormond Street Hospital for Children NHS Foundation Trust Clinical Audit Team (reference number: 2689). Data were anonymised and no personally identifiable information were collected or described in this paper. We sought patient informed consent for publication.

### Sample

**Figure 1.**
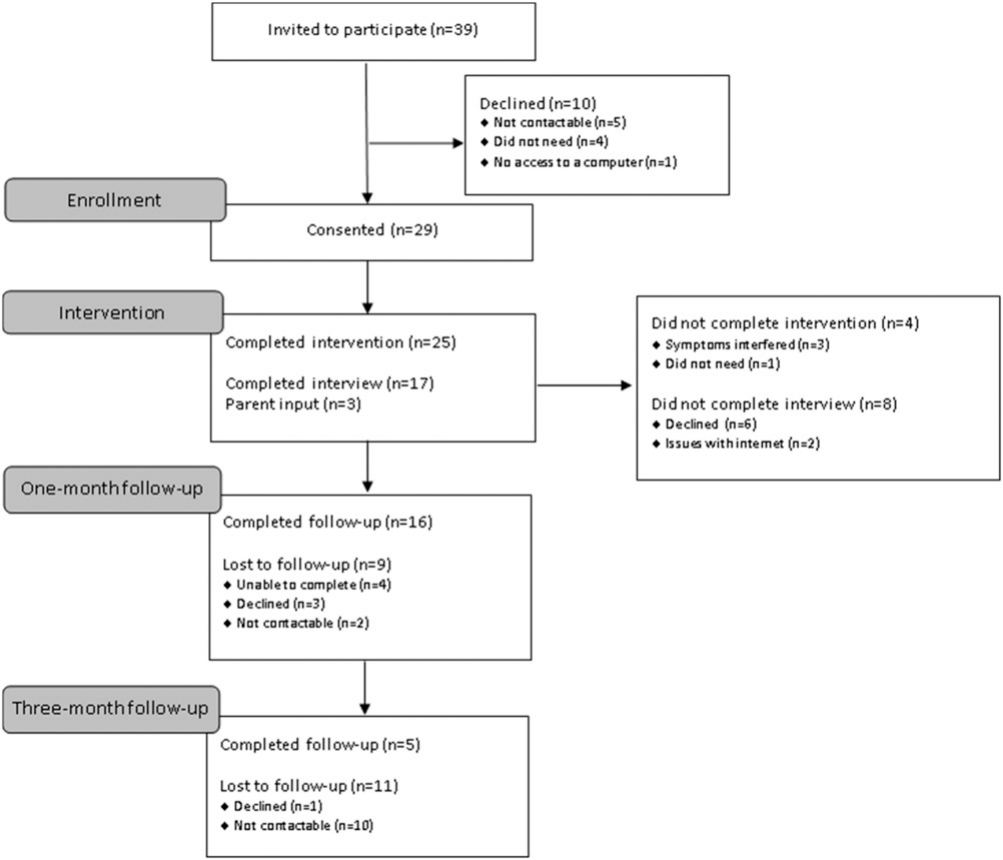
Consort diagram.

Patients aged 8–18 were recruited from waiting lists for assessment and/or treatment in a specialist mental health service in a paediatric hospital in London, United Kingdom, between March and June 2020. The patients seen in this service have complex physical and mental health needs, the majority requiring neuropsychiatric care, including Tourette syndrome (TS) and functional symptoms, as well as comorbid emotional symptoms, like depression and anxiety. Patients were excluded if they had active suicidal ideation, needed a translator, or had a chronological/developmental age below 8 years old, identified in their electronic patient records. See Figure 1 for the recruitment flowchart.

### Intervention

The 20-to-30-minute digital growth mindset SSI was based on a pre-existing US-based intervention (Schleider & Weisz, 2018). The intervention was self-administered and accessed through the internet via the Qualtrics platform, where materials were read or listened to. The intervention covered the four B.E.S.T. elements of SSIs (Schleider et al., 2020):

(a) Brain science to normalise concepts (teaching about neuroplasticity to highlight the malleability of thoughts, feelings, and behaviours, including a story about Phineas Gage (Macmillan, 2000));
(b) Empower young people to a “helper/expert” role (opportunity to give advice to other young people);
(c) Saying-is-believing exercises to solidify learning (internalisation of learning through open text);
(d) Testimonials and evidence from valued others (research about and stories of young people who overcame difficulties).

We adapted the intervention to ensure it was appropriate for a UK sample. A focus group discussion was conducted with the research team, which included clinical psychologists, psychiatrists, and researchers (*n* = 11). The discussion explored four topics: (a) strengths of the intervention; (b) potential impact on patients with depression and anxiety; (c) adaptations needed to suit British patients; and (d) other improvements. We piloted the intervention with two patients who were accessing treatment in the service and sought feedback on their experiences of the intervention. Based on the focus group discussion and pilot, we added British language, audio narration, and stories of young people of different ages.

### Measures

Demographic and clinical information, including age, sex, ethnicity, physical health problems, and presenting mental health difficulties and neurodevelopmental disorders were collected from electronic patient records at baseline.

#### (i) Primary outcome

Symptoms of depression and anxiety were assessed at baseline, 1-month follow-up, and 3-month follow-up using the young person self-report Revised Child Anxiety and Depression Scale (RCADS) (Chorpita et al., 2000). Comprised of 47-items, responses are rated on a four-point scale from 0 (‘never’) to 3 (‘always’).

#### (ii) Secondary outcomes

Perceived primary control was assessed at baseline, post-treatment, 1-month follow-up, and 3-month follow-up using the Perceived Control Scale for Children (PCSC) (Weisz et al., 2001). Statements (e.g. ‘I can do well on tests at school if I study hard’) are rated on a four-point scale from 0 (‘very false’) to 3 (‘very true’).

The 20-item Secondary Control Scale for Children (SCSC) was used to measure perceived secondary control (Weisz et al., 2010) at baseline, post-treatment, 1-month follow-up, and 3-month follow-up. Positive and negative statements are rated on a four-point scale from 0 (‘very false’) to 3 (‘very true’).

Personality mindset was assessed using the 3-item Implicit Personality Theory Questionnaire (IPT-Q) which captures beliefs about the malleability of personality (Yeager et al., 2013) and used as a manipulation check. It was administered at baseline, post-treatment, 1-month follow-up, and 3-month follow-up. Responses are rated on a six-point scale from 1 (‘really disagree’) to 6 (‘really agree’).

#### Interviews

Semi-structured interviews with patients with/without their carer/parent(s) based on patient preference were conducted and audio-recorded. The interview schedule (see Supplemental materials) was developed to explore: (a) what patients learnt; (b) what patients enjoyed and disliked; (c) perceived impact of the intervention; and (d) possible improvements. Interviews were conducted by BCFC and supervised by NM, a specialist qualitative researcher.

#### Procedure

We telephoned carers/parents, and in discussion with the young person, offered an appointment to receive the intervention. Carers/parents were informed that it was optional and their decision regarding participation would not impact their child’s clinical care or position on the waiting list. Interested families were emailed information about the intervention and informed consent was sought. Consented families were emailed baseline measures to complete before the appointment.

The appointments were planned to be face-to-face in hospital. However, due to restrictions in response to coronavirus disease (COVID-19), appointments took place through telephone/video call. Prior to the appointment, families were emailed the link to the intervention. At the start of the appointment, we explained what would happen during the session and confirmed whether patients could access the intervention online. Patients were asked to complete the intervention with/without their carer/parent depending on their preference and/or need. The researcher (BCFC) was available throughout for support. Upon intervention completion, patients were asked to complete post-treatment measures that were emailed to families at the start of the appointment. We asked patients and carers/parents to participate in an optional 15-to-30 minute telephone/video call interview about their experiences of the intervention. Interested patients were given a 30-minute break before the interview.

The appointment lasted approximately one-to-two hours depending on whether an interview was conducted. All appointments were supervised by SDB and RS, clinical psychologists. We emailed families follow-up measures one- and 3-months after the appointment.

### Analysis

#### (i) Statistical analysis

We conducted all analyses on SPSS statistical analysis software (V.25, IBM). We calculated the mean recruitment, treatment completion, and retention rates, mean subscale and total scores of the RCADS, and mean total scores of the PCSC, SCSC, and IPT-Q at baseline, post-treatment, 1-month follow-up, and 3-month follow-up. We conducted a Wilcoxon Sign-Rank Test to compare changes in scores between baseline and post-treatment/follow-ups. As a feasibility pilot, we present 95% confidence interval (CI) estimations instead of *p*-values (Lancaster et al., 2004). We calculated standardised effect size (Cohen’s d) estimations with the formula used by G*Power (Faul et al., 2007).

#### (ii) Qualitative analysis

Audio recordings of interviews were transcribed verbatim. We conducted thematic analysis (Braun & Clarke, 2006) to explore patients’ experiences of the intervention on NVivo (V.12, QSR International Pty). BCFC identified initial codes based on a sub-sample of transcripts and developed a coding frame to analyse further transcripts. This involved grouping related codes and developing themes to capture broader concepts. Themes were refined iteratively throughout the analytic process, and their conceptual coherence was discussed extensively amongst the research team (BCFC, SDB, NM, and RS). The team’s diverse perspectives (clinicians and researchers in paediatric mental health and a qualitative methodologist) were considered in these analytic discussions to enhance reflexivity (Barry et al., 1999). We used post-interview reflective field notes made by BCFC and frequent team discussions during data collection to enhance the validity of analysis (Miles & Huberman, 1994).

## Results

### Sample characteristics

Fourteen (56%) patients were male and 21 (84%) were White. Patients reported different physical health problems including pain and neurological conditions. The most common presenting mental health and neurodevelopmental difficulties included TS (17, 68%), generalised anxiety (16, 64%), autism spectrum disorder (ASD; 9, 36%), functional symptoms (8, 32%), and depression (7, 28%). Twenty-one patients (84%) presented with co-occurring difficulties. See Table 1 for more details.

**Table 1.** Baseline characteristics of patients who completed intervention and interviews.

	Completed intervention (*n* = 25)	Completed interviews (*n* = 17)
	Median	Median
Age	14	13
	*n*	*n*
Sex
Male	14	12
Female	11	5
Ethnicity
White	21	15
Asian	3	1
Mixed (White and Asian)	1	1
Physical health problems
Diabetes	1	0
Respiratory	2	2
Neurological	3	1
Pain	2	1
Other	8	3
Presenting mental health and neurodevelopmental difficulties*
Generalised anxiety	16	10
Panic	2	0
Social phobia	5	2
Specific phobia	2	2
Obsessions/compulsions	5	4
Depression	7	4
Tourette syndrome	17	15
Functional symptoms	8	3
Attention deficit hyperactivity disorder	3	5
Autism spectrum disorder	9	4
Learning disability	3	2
Multiple mental health and neurodevelopmental difficulties	21	14

*Patients may have multiple presenting difficulties and so frequency may exceed total sample.

Thirteen patients scored above clinical threshold for the RCADS subscales and total scores at baseline (see Supplementary Table 1); the most prevalent being separation anxiety (13, 52%), depression (12, 48%), and panic (11, 44%). See Table 2 for the mean RCADS subscales and total scores, PCSC, SCSC, and IPT-Q.

**Table 2.** Mean scores of outcome measures at each time-point, and change scores, standardised effect sizes, and 95% confidence intervals between baseline and other time-points.

	Mean (SD)								Change					
	*n*	Baseline	*n*	Post-treatment	*n*	One-month follow-up	*n*	Three-month follow-up	Baseline versus post-treatment		Baseline versus 1-month follow-up		Baseline versus 3-month follow-up	
									*Z*	d (95%CI)	*Z*	d (95%CI)	*Z*	d (95%CI)
RCADS separation anxiety	25	62.5 (14.5)			16	61.8 (17.1)	5	61.4 (17.8)			−.402	0.04 (−0.59, 0.67)	−1.069	0.07 (−0.89, 1.03)
RCADS generalised anxiety	25	53.0 (14.7)			16	54.1 (16.6)	5	46.2 (15.7)			−.670	−0.07 (−0.70, 0.56)	−.535	0.45 (−0.52, 1.42)
RCADS panic	25	58.6 (16.9)			16	56.6 (15.4)	5	53.8 (16.5)			−.245	0.12 (−0.51, 0.75)	−1.069	0.29 (−0.67, 1.25)
RCADS social phobia		54.5 (14.0)			16	58.8 (13.3)	5	55.0 (17.1)			−.079	−0.31 (−0.94, 0.32)	−.184	−0.03 (−0.99, 0.93)
RCADS obsessions/compulsions	25	50.3 (16.2)			16	51.6 (15.7)	5	47.4 (16.8)			−.358	−0.08 (−0.61, 0.55)	−.577	0.18 (−0.78, 1.14)
RCADS depression	25	60.4 (14.8)			16	63.6 (16.1)	5	58.0 (20.9)			−.594	−0.21 (−0.84, 0.42)	−.271	0.13 (−0.83, 1.09)
RCADS total anxiety	25	57.0 (16.2)			16	58.4 (17.2)	5	54.4 (20.5)			−.315	−0.08 (−0.71, 0.55)	−.136	0.14 (−0.82, 1.10)
RCADS total anxiety and depression	25	58.1 (16.2)			16	59.9 (17.3)	5	55.6 (22.1)			.000	−0.11 (−0.74, 0.52)	−.674	0.13 (−0.83, 1.09)
PCSC	24	50.3 (11.7)	25	51.3 (13.2)	16	47.5 (14.1)	5	52.4 (10.9)	−.732	−0.08 (−0.64, 0.48)	−.909	0.22 (−0.42, 0.86)	−.271	−0.19 (−1.15, 0.77)
SCSC	25	26.6 (13.6)	25	27.2 (15.0	16	22.6 (14.5)	5	35.6 (19.8)	−.521	−0.04 (−0.59, 0.51)	−.104	0.28 (−0.35, 0.91)	−2.023	−0.53 (−1.50, 0.44)
IPT-Q	23	11.7 (4.6)	24	9.2 (4.7)	16	10.8 (3.5)	5	6.4 (3.8)	−2.943	0.54 (−0.04, 1.12)	−1.264	0.22 (−0.42, 0.86)	−1.461	1.26 (0.24, 2.28)

### Feasibility

#### (i) Recruitment, treatment completion, and retention rates

Thirty-nine patients were contacted and 29 (74%) consented. Of these, 25 (86%) completed the intervention. Seventeen patients and three carers/parents completed the interviews. Sixteen (55%) and 5 (17%) patients completed 1-month and 3-month follow-up measures, respectively. See Figure 1 for the recruitment flow.

### Impact

#### (i) Outcome measures

We found moderate improvement in IPT-Q (d = 0.54) but none in PCSC (d = −0.08) and SCSC (d = −0.04) at post-treatment. Negligible improvements were seen across measures at 1-month follow-up (see Table 2). We found large improvement in IPT-Q (d = 1.26), moderate improvements in generalised anxiety (d = 0.45) and SCSC (d = 0.53), and small improvements in panic (d = 0.29), obsessions/compulsions (d = 0.18), and PCSC (d = 0.19) at 3-month follow-up.

We found no difference in treatment effects between patients who met the clinical threshold on the RCADS scores at baseline and the full sample, and therefore did not report this.

### Acceptability

The thematic analysis produced findings about patients’ experiences of the intervention within three clusters presented below: Overall accessibility and interest; specific components of intervention; and potential perceived impact of intervention.

#### (i) Overall accessibility and interest

Almost all patients reported completing the intervention independently within 10-to-30-minutes. Many patients described never having come across a similar intervention, and most reported enjoying completing it and found it clear and understandable. Some stated that the visually attractive slides, which included pictures and graphs, maintained their focus and motivation to complete the intervention.

*“I enjoyed how for each slide they gave you something you could see as well, like an actual image. It made it very easy to visualise and understand… I could actually pay attention to what I’m reading.” – PID 17 (male, 15, functional symptoms)*

Some adolescent male patients with varying mental health difficulties said they did not find the intervention interesting; no further detail was provided when prompted to elaborate. These patients were also less responsive overall in the interview.

#### (ii) Specific components of intervention

##### Audio narration

Many patients highlighted that having the option of reading or listening to a narration of the slides was helpful for sustaining attention, especially for those with attention or learning disabilities. The clear narration facilitated better understanding of the content and made their experience enjoyable.

##### Research

Inclusion of research on other young people’s experiences was deemed helpful by many patients because it normalised their own experiences. Most said they were aware of the possibility of overcoming their difficulties but seeing it through research findings solidified their beliefs.

##### Neuroplasticity

Many reported being particularly interested in learning about neurons and their link with personality, thoughts, feelings, and behaviours, especially the Phineas Gage story because of its gory nature. Patients expressed that although a complex topic, the content was digestible because of the clear explanations. One autistic patient who disliked human anatomy reported feeling discomfort in reading about neurons, which impaired their concentration.

##### Other young people’s stories

Most patients described other young people’s stories as valuable and relatable which promoted identification, despite experiences not being identical. Many spoke about how the stories importantly emphasised how others experienced adversity too.

*“You know that you’re not the only one who has problems. That other people suffer too.” – PID 12 (male, 13, TS)*

Some reported learning strategies that others used to overcome their difficulties and manage their mood. Some patients noted that the strategies seemed easy to implement, while others wanted more clarity. A few patients noted the stories felt inauthentic. This, in addition to not being able to relate to stories from older adolescents, reduced the relatability of the stories for some. Many patients expressed wanting greater diversity in young people’s ages and difficulties to make the stories more relatable. Some felt the stories of peer difficulties at school did not capture the variety of problems they faced such as general interactions with friends or exams.

##### Giving advice

Most patients reported feeling motivated to advise other young people going through difficulties in the open text; they felt proud they could potentially help others. Some described this process as helpful in consolidating their learning from the intervention.

*“I know that other kids would be seeing this and then they’d know how I felt about it. I felt pretty good because I know that they would be looking at it and some of them might even try it.” – PID 9 (male, 12, TS)*

However, some older autistic patients recounted giving advice as challenging and overwhelming; they struggled to understand the questions, comprehend what other young people may be thinking and feeling, relate to the example scenarios, and feel that their advice was sound.

##### Wanting more

Some patients and their carers/parents thought the intervention could have included more on changing negative beliefs; they described seeing the value of learning about growth mindset but were unsure about how to change embedded beliefs. One carer/parent suggested including reflective journals and mind maps so patients could take them away after the intervention to practice.

#### (iii) Potential impact of intervention

When asked if they would recommend the intervention to another young person going through similar difficulties to themselves, all patients endorsed the intervention and recognised its potential benefit irrespective of whether they found it helpful themselves.

##### Hope of change

Many patients described feeling mistreated by others the past, which made them feel sad, confused, and angry. These patients reported that the intervention instilled hope that these young people could change.

*“At my college, when I first started, I had loads of friends. Gradually they all turned against me because of my illness. Before [the research], I would’ve stuck to the opinion that I don’t think people can change. Clearly, they can.” – PID 6 (female, 17, autistic, functional symptoms)*

Some patients also described being hopeful of change in themselves, reporting an enhanced recognition of the fluidity of their own predicament, thoughts, and feelings. Some referred to the fact that other young people could feel better as evidence for the possibility of their own change.

*“It gives you a sense of what other people are going through and that they’ve changed and that you can change too.” - PID 12 (male, 13, TS)*

However, a few patients spoke about feeling simultaneously hopeful and doubtful. Although encouraging, patients wondered if the impact of the intervention was more fleeting than permanent as they anticipated difficulty in applying their learning in daily life.

*“As much as I find it easy after reading it, when I actually face some situations like that, I won’t be able to hold onto it in the moment”. – PID 17 (male, 15, functional symptoms)*

##### New perspective

As a result of the intervention, some patients described acknowledging that their thoughts can be unhelpful, and problems can be framed positively. This extended to a deeper awareness that their thoughts, feelings, and behaviours are malleable, and may inform responses to future problems. One participant expressed that this shift in perspective made them confident in their own ability to tackle difficulties.

*“Say I go to someone’s party and I don’t really know anyone. I might feel more confident speaking to people now.” – PID 26 (male, 15, TS)*

##### Reflection

For many, hearing others’ stories brought back painful memories of their own difficulties. However, some saw this as an opportunity to reflect on past responses to problems and how they can respond adaptively in future situations. A few reflected on how they could apply their learning to different contexts, such as family conflict and the pandemic.

*“Is there really a problem or am I just being negative about the way I think about it? I think it would help me if I was talking to my parents or my brother or sister because we’re all stuck at home at the moment.” – PID 27 (female, 13, TS)*

##### Pathway to overcoming difficulties

A few patients described the intervention as a ‘first step’ in overcoming their difficulties; being cognizant of the possibility of change may promote recovery. Regardless of the presence of perceived immediate benefit, some expressed hope for long-term benefit. Another reported that completing the intervention may have made them more open to other treatments.

## Discussion

The findings from this pilot suggest that the adapted digital growth mindset SSI is feasible and acceptable for young people with physical and mental health needs on waiting lists in a paediatric hospital mental health service. High recruitment and treatment completion rates demonstrate patients are willing to receive the intervention as part of a remote appointment. Qualitative interviews suggest that most patients enjoyed completing the intervention because of the visuals, content, and computer-guided format. Most patients reported completing the intervention independently, which highlights the accessibility and feasibility of the intervention for young people. The option to complete the intervention with carer/parent support also demonstrates the possibility of flexible delivery based on individual patient needs. This supports previous findings that suggest digital interventions may be more accessible to young people than traditional treatments (Hollis et al., 2017).

We were unable to replicate published treatment effects, but this may be because our sample did not meet clinical threshold on the RCADS at baseline at a group level; this may have made it difficult to identify meaningful improvement. Previous trials identified strongest effects (d = 0.32–0.60 for depression, d = 0.28–0.33 for anxiety, and d = 0.24–0.27 for perceived control; Schleider & Weisz, 2018) among samples who had clinical levels of depression and anxiety (Schleider & Weisz, 2018; Schleider, Mullarkey, et al., 2021). However, the descriptive statistics indicate small to moderate effect size improvements in personality mindset, symptoms of depression and anxiety, and perceived control suggesting potential value for young people with complex needs. Facilitating the development of growth mindset that is driven by the possibility of change via self-determination and hope (Dweck, 2008) in young people with physical and mental health symptoms may improve long-term outcomes, as hope is a significant predictor of depression and anxiety in chronic illness (Rasmussen et al., 2017). As our qualitative interviews suggest, developing a growth mindset whilst on waiting lists may be an important precursor for preparing young people for psychological interventions through increased motivation.

There are limitations to this study. The self-administered nature of the intervention may have inadvertently restricted our sample to only including patients who had fewer impairing symptoms; three patients did not complete the intervention due to symptom interference like attention difficulties. Conducting interviews immediately after treatment allowed us to capture experiences of the intervention without recall problems but restricted our ability to explore the intervention’s perceived longer-term impacts. Social desirability may have influenced young people’s responses to questions about the intervention as the same researcher administered the interview and collected research data. There was low follow-up retention which may be explained by respondent burden from completing long measures such as the RCADS. Although we demonstrate positive intervention effects at follow-up, attrition may have skewed intervention effects and only captured responses from patients who experienced improvements in outcomes. The small sample at follow-up may have also limited analysis of outcomes and needs to be accounted for when interpreting effect sizes. However, this should be considered within the restraints of conducting paediatric clinical research during the pandemic (Stiles-Shields et al., 2020).

Future research should use larger samples and control groups to isolate and assess the intervention’s effectiveness in improving outcomes in paediatric samples, and nested qualitative studies in follow-ups of larger randomised controlled trials to evaluate the impact of the intervention after young people leave waiting lists (e.g. to start treatment). Continuing to conduct research using mixed methods may add further value beyond our findings and explore mechanisms of change and its implementation in child and paediatric mental health services.

Despite the limitations, the potential integration and use of digital SSIs in specialist paediatric mental health services is promising. The representative nature of the sample indicates that a brief intervention with little-to-no therapist input can be easily delivered remotely to paediatric patients. This pragmatic pilot suggests the highly accessible intervention can be offered to patients on waiting lists for mental health treatment. This is relevant to children services during COVID-19, as we have seen an uptake in technology use to maintain service provisions (Ching et al., 2021; Sharma et al., 2020). A recent trial found that a digital SSI improved mental health outcomes of students during COVID-19 (Wasil et al., 2021) suggesting that SSIs are especially useful when access to care is difficult.

The use of mixed methods provided rich data about important patient and intervention factors and areas for modification. Intervention ‘ingredients’ deemed important by patients and carers/parents were elicited by the interviews, such as the online interface, delivery options, and relatability of stories. Patients suggested varying the age and problems in the stories of young people, covering explicit strategies to identify and change negative thoughts, and providing takeaway materials to promote application of learning.

Deeper understanding of the acceptability and impact of the intervention in autistic patients is necessary as highlighted by differences in experiences identified in our qualitative findings. A recent study evaluating a longer growth mindset intervention in young people with mild to borderline intellectual disabilities reported high satisfaction in the intervention (Verberg et al., 2021), though no qualitative data was collected. This is vital as young people with communication difficulties may be more likely to endorse a fixed mindset (Brooks & Goldstein, 2013; Verberg et al., 2019).

## Conclusion

The digital growth mindset SSI is feasible, acceptable, and potentially useful for young people with complex physical and mental health needs on waiting lists for mental health treatment in a paediatric hospital. This pilot study integrates data on patients’ diverse experiences and views of the intervention, providing useful implications for clinical services and intervention modification. Further robust research is warranted to examine the intervention’s long-term effectiveness and mechanism of change.

## Supplemental Material

sj-pdf-1-ccp-10.1177_13591045221105193 – Supplemental Material for Growth mindset in young people awaiting treatment in a paediatric mental health service: A mixed methods pilot of a digital single-session intervention Click here for additional data file. Supplemental Material, sj-pdf-1-ccp-10.1177_13591045221105193 for Growth mindset in young people awaiting treatment in a paediatric mental health service: A mixed methods pilot of a digital single-session intervention by Brian CF Ching, Sophie D Bennett, Nicola Morant, Isobel Heyman, Jessica L Schleider, Kate Fifield, Sophie Allen and Roz Shafran in Polymers and Polymer Composites
