# Supplementary material for: Growth mindset in young people awaiting treatment in a paediatric mental health service: A mixed methods pilot of a digital single-session intervention
Source: Clin Child Psychol Psychiatry. 2022 Jun 1;28(2):637–53. doi: 10.1177/13591045221105193 (PMC10018056; doi:10.1177/13591045221105193)
Supplement: sj-pdf-1-ccp-10.1177_13591045221105193 – Supplemental Material for Growth mindset in young people awaiting treatment in a paediatric mental health service: A mixed methods pilot of a digital single-session intervention [file sj-pdf-1-ccp-10.1177_13591045221105193.pdf]

Supplementary Table 1

Number of patients who met clinical threshold for the Revised Children's Anxiety and Depression Scale (RCADS) subscales at baseline. Some participants met clinical threshold for multiple subscales.

	n
Separation anxiety	13
Generalised anxiety	3
Panic	11
Social phobia	5
Obsessions/compulsions	6
Depression	12
Total anxiety	8
Total anxiety and depression	8
